# Improvement in Drainage Systems

**Published:** 1919-07-19

**Authors:** 


### HOSPITAL CONSTRUCTION: THE NEW ERA.

### Improvement in Drainage Systems.

ONE of the most striking facts in the constructional history of the past fifty years is to be found in the world of drainage. We refer to the introduction of a sort of code of sanitation which is almost as solemnly venerated at the decalogue and much more solemnly observed. Probably the rigidity of our existing drainage code is due to two causes. One is the thoroughness of the sanitary awakening which took place early in the last quarter of the nineteenth century, and the other is the establishment of by-laws and of sanitary officers throughout the country. The by-laws, to be sure, would have been of little service by themselves. If "the law is an ass" the by-law is—well, leave it at that.

But the wonderful fact is that though these elusive by-laws are carried into effect by countless surveyors and inspectors, the practice throughout the country is marvellously uniform. This we owe, no doubt, to the very sound theoretical and practical basis on which the sanitary renaissance of the 'eighties was founded. The main principles then enunciated, through-ventilation, absolute watertightness of drains and pipes, absolute air-tightness, incontestable "seal" in all traps, and absolute cleanliness of traps were laid down as law, and as law they have remained. So entirely are they beyond dispute, and so rigid is the adherence to the "jots and tittles" of this drainage code, that no man dares to use his inventive faculty in the way of minor improvements. We accept the whole body of the law, and nc Pharisee could be more averse than we moderns are from the very idea of any origination in the way of departure. Sometimes this conservatism goes strange lengths and rather stupid lengths. Some wise man a quarter of a century ago condemned all "wash-out" closets, and decided that the "wash-down" type should be substituted for them. We suppose that as a result of sanitary inspection many hundreds of the "wash-out" type have found their way to the dust-bin, and most of them deserved their discharge. But there is at least one make of "wash out" with a front "weir" which is a most excellent and cleanly article; and, as this make of closet alone gives an opportunity for the kind of inspection which is essential in a well-managed nursery, it is not too much to say that one closet at least of this type should be in every family house. The only instance we can remember of any stalwart attempt to stem the barriers of the sanitary code as accepted was the effort of the late Mr. Norman Shaw, R.A., to popularise a 2½ in. or 3 in. soil pipe with a trap at its foot! As this private heresy was, to speak honestly, an unhygienic one, no tears need be shed over its failure. Strangely enough the valve-closet, which was condemned for its association with the "D" trap, and perhaps for its cousinship to the pan-closet (a now extinct and prehistoric memory), won its way back into favour. This recovery was due partly to the excellent type or pattern which the inventor of the "anti-D" trap brought on to the market, but mostly, we believe, to the undoubted fact that some users of closet apparatus entirely and rightly decline to advertise their private actions by a loud, very public, and quite unmistakable sound signal.

Our sanitary code being so good, it is quite well that it should be faithfully observed and loyally kept. But is this enough to satisfy a progressive generation? Surely it is time that we saw research at work, invention at play. Sewage treatment and sewage disposal problems have by no means stagnated. The improvements made since the day when the old-world cesspool with its night-soil horrors was still regarded as an evil that had to be tolerated are numerous, but the household system, from the syphon trap upwards, remains stationary. What is the meaning of this? We attribute it to nothing more or less than the sanctity of the existing code. Nobody dares to invent. If an inventor were to make a discovery which led to an invention running counter to one of our unwritten or written laws of house drainage, he would meet with such opposition and hindrance that it would be scarcely worth his while to press his point. We are rash enough to venture on this general and sweeping statement in the hope that some reader, if he is aware of any novelty which in the past ten or twenty years has bearded the by-laws or rebuffed the inspectors, will communicate the facts.

Looking at hospitals in particular, we remind ourselves that the Americans are apt to make fun of the "old English sanitary tower." They speak of it rather as if it belonged to the Edwardian age of the fourteenth century instead of to the modern Edwardian. But which of us would dare to design a hospital without it? True, the "Plenum" enthusiasts claimed to do without its cross-ventilated "cut-off," perceiving that so long as their plenum ventilation would work the "cut-off" was unnecessary. But where natural ventilation prevails, it seems to most of us impossible to dispense with the form of plan generally and traditionally adopted. At the same time, there is no doubt that the "Tower" is an expensive and very awkward appendage to a ward, and if there were any way of securing healthy and inoffensive sanitary accommodation without this method of segregation, it would be a good day for the hospital planner.

To-day is the day of thought. Let us think about drainage as about everything else. Nothing is good merely because it exists or even because it *has* been good. Good leads to better, and better to best; so let us worry our brains over this household sanitation problem in the hope that excellent as our sanitary science is to-day, it may be found in a few years to be as obsolete as the first-class drainage of 1850.

Previous articles appeared on Jan. 25, Feb. 8, March 1, 15, 29, April 19, May 17, and June 14, p. 296.

